# Simm530, a novel and highly selective c-Met inhibitor, blocks c-Met-stimulated signaling and neoplastic activities

**DOI:** 10.18632/oncotarget.9349

**Published:** 2016-05-13

**Authors:** Ying Wang, Zhengsheng Zhan, Xifei Jiang, Xia Peng, Yanyan Shen, Fang Chen, Yinchun Ji, Weiren Liu, Yinghong Shi, Wenhu Duan, Jian Ding, Jing Ai, Meiyu Geng

**Affiliations:** ^1^ Division of Anti-Tumor Pharmacology, State Key Laboratory of Drug Research, Shanghai Institute of Materia Medica, Chinese Academy of Sciences, Shanghai, P.R.China; ^2^ Department of Medicinal Chemistry Shanghai Institute of Materia Medica, Chinese Academy of Sciences, Shanghai, P.R.China; ^3^ Department of Liver Surgery, Liver Cancer Institute, Zhongshan Hospital, Fudan University, Shanghai, P.R.China; ^4^ Key Laboratory of Carcinogenesis and Cancer Invasion of Ministry of Education, Shanghai, P.R.China

**Keywords:** c-Met, kinase inhibitor, Simm530

## Abstract

The aberrant c-Met activation has been implicated in a variety of human cancers for its critical role in tumor growth, metastasis and tumor angiogenesis. Thus, c-Met axis presents as an attractive therapeutic target. Notably, most of these c-Met inhibitors currently being evaluated in clinical trials lack selectivity and target multiple kinases, often accounting for the undesirable toxicities. Here we described Simm530 as a potent and selective c-Met inhibitor. Simm530 demonstrated >2,000 fold selectivity for c-Met compared with other 282 kinases, making it one of the most selective c-Met inhibitors described to date. This inhibitor significantly blocked c-Met signaling pathways regardless of mechanistic complexity implicated in c-Met activation. As a result, Simm530 led to substantial inhibition of c-Met-promoted cell proliferation, migration, invasion, ECM degradation, cell scattering and invasive growth. In addition, Simm530 inhibited primary human umbilical vascular endothelial cell (HUVEC) proliferation, decreased intratumoral CD31 expression and plasma pro-angiogenic factor interleukin-8 secretion, suggesting its significant anti-angiogenic properties. Simm530 resulted in dose-dependent inhibition of c-Met phosphorylation and tumor growth in c-Met-driven lung and gastric cancer xenografts. And, the inhibitor is well tolerated even at doses that achieve complete tumor regression. Together, Simm530 is a potent and highly selective c-Met kinase inhibitor that may have promising therapeutic potential in c-Met-driven cancer treatment.

## INTRODUCTION

The oncogene *MET* encodes the receptor tyrosine kinase for hepatocyte growth factor (HGF) [1–4]. Activation of the c-Met pathway triggers a unique genetic program, known as the “invasive growth”, which physiologically underlies tissue morphogenesis. Aberrant execution of this program has been associated with neoplastic transformation, invasion and metastasis [5–8].

Abnormal c-Met activation has been frequently observed in a variety of human solid tumors and hematologic malignancies, either as a consequence of gene amplification, mutation, or rearrangement, transcriptional up-regulation as well as autocrine or paracrine ligand stimulation [5–8]. Furthermore, HGF and c-Met have been implicated in regulation of tumor angiogenesis through the direct pro-angiogenic properties of HGF or through the regulation of pro-angiogenic factors secretion [9–11]. Increasing evidence suggests that both c-Met and HGF elevations have been associated with poor clinical outcomes [5–8]. Moreover, over-activation of HGF/c-Met axis has been linked to acquired or *de novo* resistance to targeted therapies, such as EGFR, B-Raf and HER-2 inhibitors [12–15]. Thus, c-Met axis has emerged as an attractive target for therapeutic medication of cancer.

Over the past decade, in spite of a remarkable number of c-Met inhibitors undergoing preclinical and clinic assessment, none of them has been approved for clinical use [6, 16–22]. Notably, most of these c-Met inhibitors lack selectivity and inhibit multiple kinases, which would increase the risk of unwanted “off-target” toxicities. More importantly, in the era of precision medicine, a highly specific c-Met inhibitor would be more suitable to fulfill the specific treatment need for sub-population of c-Met-driven cancer and serve as a “clean” component for combination strategies against c-Met-mediated drug resistance. Thus, more selective c-Met inhibitors are required.

Here, we reported a highly selective and potent c-Met inhibitor, Simm530. Simm530 exhibits sub-nanomolar level enzymatic potency and is highly specific for c-Met with more than 2,000-fold selectivity over a large panel of 282 human kinases. Simm530 potently blocked c-Met phosphorylation and the downstream signaling in c-Met over-activated cancer cell lines. As a result, it inhibited c-Met-stimulated cellular events in tumor cells and primary endothelial cells. Moreover, Simm530 exhibited significant antitumor activity in c-Met-driven xenograft models at well tolerated doses. All these findings promise Simm530 as a potential candidate for c-Met-driven human cancers.

## RESULTS

### Simm530 is a potent and highly selective c-Met inhibitor

**Figure 1:**
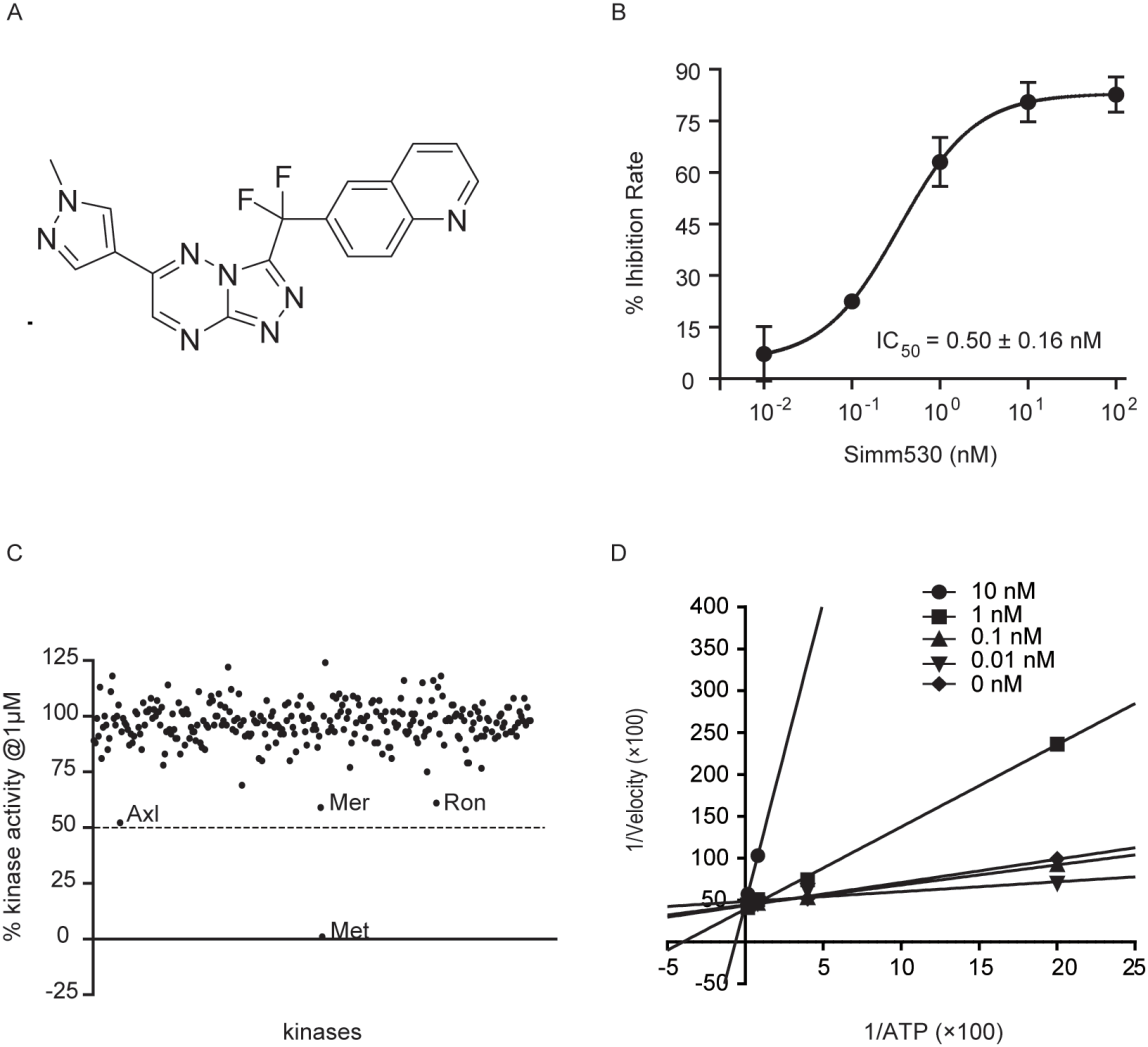
Simm530 is a potent, highly selective and ATP-competitive inhibitor of c-Met **A.** The chemical structure of Simm530. **B.** The inhibition curve of Simm530 on c-Met kinase activity. **C.** Kinase-selectivity profile of Simm530 on 282 human protein kinases. **D.** Lineweaver-Burk plot demonstrating the ATP-competitive inhibition of c-Met kinase activity by Simm530.

Simm530 was initially identified as a potent c-Met kinase inhibitor with an IC_50_ value of 0.50 ± 0.16 nM using an ELISA assay with recombinant c-Met kinase protein (Figure 1A, 1B). Accordingly, we were prompted to investigate whether this potency was specifically against c-Met. Simm530 was profiled against a panel of 282 human kinases, including c-Met family member, Ron, and c-Met homologous, Axl kinase family (Axl, Tyro3, c-Mer). Compared to its high potency against c-Met, Simm530 exhibited more than 2,000-fold less potency against these tested kinases, with inhibitory rate less than 50% at 1 μM (Figure 1C), indicating that Simm530 is a highly selective c-Met inhibitor.

As most kinase inhibitors to date are ATP competitive, we examined whether Simm530 functions in a similar manner. The inhibitory potency of Simm530 on c-Met kinase activity was evaluated with introducing increasing ATP concentration. Lineweaver-Burk plot for c-Met inhibition by Simm530 with respect to the ATP concentration showed all the curves intersecting the y-intercept at zero, which indicates a competitive mechanism of inhibition (Figure 1D). Thus, Simm530 is a potent, highly selective and ATP-competitive inhibitor of c-Met.

### Simm530 inhibits c-Met phosphorylation and its downstream signaling pathways

**Figure 2:**
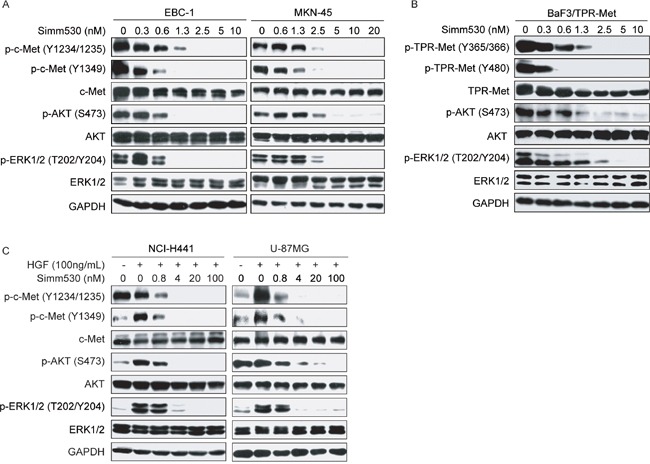
Simm530 suppresses c-Met phosphorylation and signal pathways in various cells **A**&**B.** EBC-1, MKN-45 (A) and BaF3/TPR-Met cells (B) treated with increasing concentrations of Simm530 for 2 h were lysed and subjected to immunoblotting analysis. **C.** NCI-H441 and U-87MG cells were serum-deprived for 24 h prior to 2 h treatment with Simm530 following stimulation with HGF for 15 min. Then, cells were lysed and subjected to immunoblotting analysis.

Next, we investigated the cellular kinase-targeting activity of Simm530. Firstly, EBC-1 and MKN-45 human cancer cells that harbor an amplified *MET* gene, and BaF3/TPR-Met cell that stably expressing a constitutively active oncogenic version *TPR-MET* were used. Exposure to Simm530 significantly inhibited c-Met phosphorylation at the activation loop (Y1234/1235) and its COOH-terminal docking site (Y1349), with a complete abolishment at 2.5 or 5 nM in these tested cell lines (Figure 2A and 2B). Consistently, this was accompanied by a dose-dependent inhibition of phosphorylation of c-Met key downstream signaling molecules, AKT and ERK1/2 [5, 23] (Figure 2A and 2B). Similar results was recapitulated in Simm530-treated NCI-H441 human lung cancer cells and U-87MG human glioblastoma cells, which respond well to HGF stimulation (Figure 2C).

c-Met-activating mutations have been identified in human cancers and some reportedly confer resistance to certain c-Met kinase inhibitors [24–29]. Thus, Simm530 was evaluated for its ability to inhibit c-Met phosphorylation in a panel of NIH/3T3 cell lines expressing c-Met mutations, including three activation loop mutants (Y1230C, Y1230H, and Y1235D) and a P + 1 loop mutant (M1250T). As shown in Figure S1, Simm530 exhibited similar potency against c-Met M1250T, Y1235D and Y1230C mutants compared with the wild-type c-Met. However, a marked decrease in potency of Simm530 was observed against the c-Met Y1230H mutant, indicating that specific mutation of c-Met, Y1230H, is resistant to Simm530 treatment.

Together, these data strongly suggested that Simm530 inhibits both constitutive and ligand mediated c-Met activation and, in turn leading to the inhibition of downstream c-Met signaling.

### Simm530 specifically inhibits c-Met-addicted proliferation of human cancer cells with high potency

**Figure 3:**
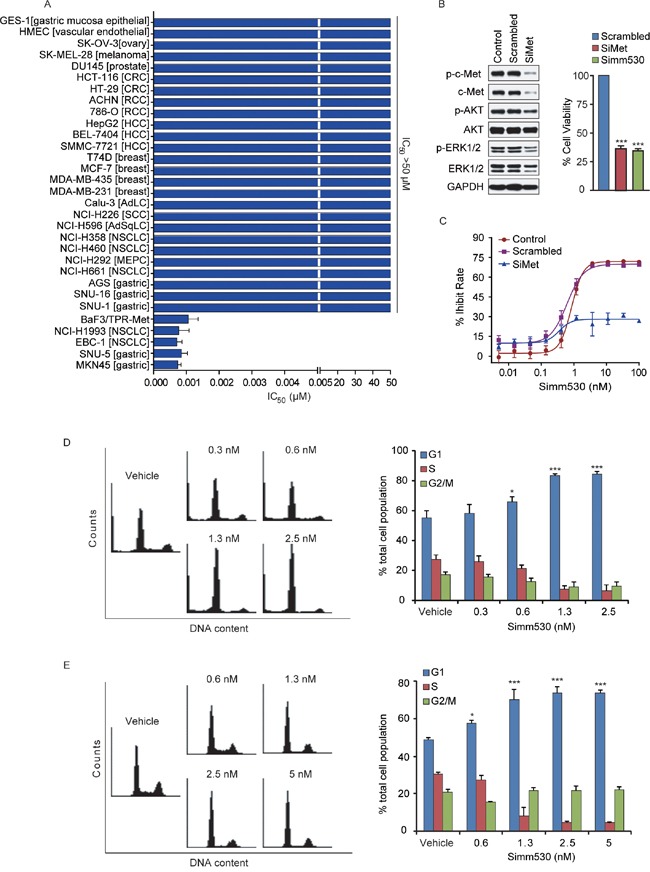
Simm530 specifically and potently inhibits c-Met-addicted proliferation of human cancer cells via G_1_/S phase arrest **A.** The anti-proliferation activity of Simm530 against a panel of tumor cell lines and normal cells originating from different tissue types was determined by a sulforhodamine B (SRB) or an MTT assay. The IC_50_ values were plotted as the means ± SD (μM) or estimated values from three separate experiments. **B.** c-Met knockdown inhibited cell growth and c-Met signaling. c-Met was disrupted using c-Met siRNAs for 96 h or Simm530 (100 nM) for 72 h in MKN-45 cells, following immunoblotting (left panel) and cell viability (right panel) analysis. Bars represent means ± SD. **C.** MKN-45 cells were treated with scramble or c-Met siRNAs for 24 h followed by increasing concentrations of Simm530 for 72 h. Cell viability were analyzed. Bars represent means ± SD. The results shown are representative of three independent experiments. **D**&**E.** Simm530 induced G_1_/S phase cell cycle arrest in c-Met-addicted human cancer cells. EBC-1 (D) and MKN-45 (E) cells were treated with indicated concentrations of Simm530 for 24 h. The percentages of cells in different cell cycle phases determined by FACS and analyzed with Modifit LT were plotted. The data shown are the mean ± SD from three independent experiments, and representative images are shown. * P < 0.05; *** P < 0.001 vs vehicle group, determined using ANOVA test.

Increased c-Met activity promotes cancer cell proliferation [5, 30, 31]. Therefore, Simm530 was evaluated on a panel of 30 human cancer cell lines and normal cells with different settings of c-Met expression/activation to determine its cytotoxic activity. As shown in Figure 3A, the IC_50_ values varied widely among the cell lines and were closely correlated with the individual c-Met status. Notably, *MET* amplification-driven cancer cells (EBC-1, NCI-H1993, MKN-45 and SNU-5) were most sensitive to Simm530 treatment, with IC_50_ values of 0.7, 0.8, 0.9 and 0.7 nM, respectively (Figure 3A, Table S1). In addition, Simm530 also significantly inhibited proliferation of BaF3/TPR-Met cells (Figure 3A, Table S1), which features c-Met addicted cell growth. In contrast, Simm530 treatment exerted little anti-proliferative effect in cells with low c-Met expression or activation (cytotoxicity IC_50_ values of >50 μM) (Figure 3A), exhibiting at least a 50,000-fold less potency than that of c-Met-addicted cancer cells. These findings, strongly suggested that Simm530 specifically inhibited c-Met-driven cell proliferation with high potency.

For further confirmation, we used siRNA knocking down c-Met protein levels in MKN-45 cells. Transfection of c-Met siRNA decreased c-Met protein expression by at least 90%. As a result, c-Met depletion yielded a pattern of pathway inhibition, and in turn cell proliferation inhibition that was remarkably similar to that observed following Simm530 treatment (Figure 3B). However, Simm530 failed to substantially inhibit the MKN-45 cell proliferation that transfected with c-Met siRNA, with no obvious inhibitory effect even at 100 nM (Figure 3C). Overall, these finding supports the conclusion that the Simm530 potently inhibited cell proliferation via specifically inhibiting c-Met.

It was previously revealed that c-Met inhibition triggers G_1_/S cell cycle arrest, and further cell proliferation inhibition as consequence [32–35]. To confirm whether the mechanism of anti-proliferative of Simm530 against c-Met-driven cancer cells was due to inhibiting c-Met signaling, cell-cycle distribution was analyzed by flow cytometry analysis. As expected, Simm530 significantly induced a G_1_/S phase arrest by dose-dependent manner in EBC-1 and MKN-45 cells (*P<0.001*) (Figure 3D, 3E). Consistently, Simm530 significantly decreased the protein level of cyclin D1 and cyclin E, two key modulators of the G_1_/S transition (Figure S2). In addition, no obvious sub-G_1_ cell population was observed upon Simm530 treatment (Figure 3D, 3E), largely excluding the occurrence of Simm530-induced apoptosis. We therefore concluded that Simm530 selectively inhibited proliferation of c-Met addicted cells via arresting cells at G_1_/S phase.

### Simm530 significantly inhibits HGF/c-Met-promoted cell metastasis phenotypes

**Figure 4:**
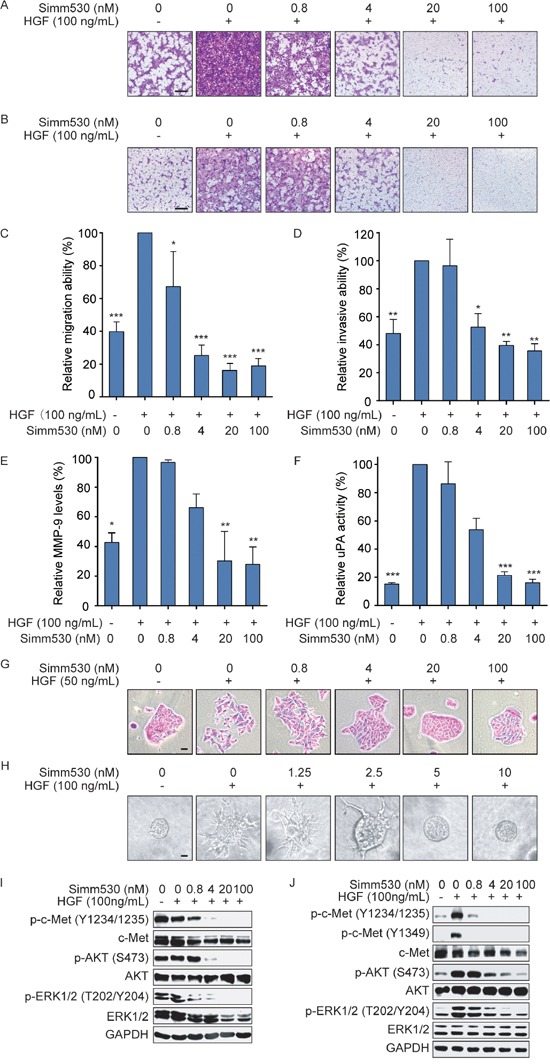
Simm530 significantly inhibits HGF/c-Met promoted cell metastasis phenotypes **A, B, C**&**D.** Simm530 suppressed HGF-induced NCI-H441cell migration (A&C) and invasion (B&D). Representative images are shown (scale bar, 100 μm). The relative migration and invasion were plotted. The data shown are the mean ± SD from three independent experiments, assuming 100% migration or invasion of cells stimulated with HGF. * P < 0.05; *** P < 0.001 vs HGF stimulating group, determined using ANOVA test. **E.** Simm530 reduced HGF-induced MMP-9 expression in NCI-H441 cells. Pre-starved NCI-H441 cells were treated with HGF and increasing concentrations of Simm530 for 24 h. Expression of pro-MMP-9 (92 kDa) was detected in same amounts of supernatant by geltin zymography and quantified by densitometric analysis using Image Lab software (Bio-Rad). The data shown are the mean ± SD from three independent experiments, assuming 100% MMP-9 activity of cells stimulated with HGF. * P < 0.05; ** P < 0.01 vs HGF stimulating group, determined using ANOVA test. **F.** Simm530 suppressed HGF-induced urokinase activity in MDCK cells. Pre-starved MDCK cells were treated with HGF and increasing concentrations of Simm530 for 24h, then uPA activity was measured. The data shown are the mean ± SD from three independent experiments, assuming 100% uPA activity of cells stimulated with HGF. *** P < 0.001 vs HGF stimulating group, determined using ANOVA test. **G.** Simm530 inhibited HGF-induced scattering of MDCK cells. Cells were grown as small colonies at low density and treated with HGF (50 ng/mL) in the presence of increasing concentrations of Simm530 for 18 to 24 h. Representative images from three separate experiments are shown (scale bars, 10 μm). **H.** Simm530 significantly inhibited HGF-stimulated invasive cell growth. The MDCK branching morphogenesis in collagen induced by HGF was inhibited by Simm530. Images were obtained 5 days after treatment. Representative images from three separate experiments are shown (scale bars, 10 μm). **I**&**J.** Simm530 inhibited c-Met signaling pathway in HGF-stimulated NCI-H441 (I) and MDCK (J) cells. Pre-starved NCI-H441 and MDCK cells were treated with increasing concentrations of Simm530 and 100 ng/mL HGF for 24 h, and then were subjected to immunoblotting analysis.

Another important function of HGF/c-Met axis activation is to promote cancer cells migration and invasion, which contribute to the tumor metastasis [5–8]. We then evaluated the effect of Simm530 in this regard. For this, NCI-H441 and Madin-Darby canine kidney (MDCK) cells, which respond well to HGF stimulation, were used. Simm530 strongly suppressed HGF-induced NCI-H441 cell motility and invasion in a dose-dependent manner and was sufficient to block the movement of most cells at a dose of 4 nM (Figure 4A, 4B, 4C, 4D). Similar results were observed in a wound-healing assay using MDCK cells (Data not shown).

Cell invasion and metastasis requires degradation of surrounding ECM. HGF/c-Met axis has been strongly implicated in promoting the production of proteinases, such as urokinase plasminogen activator (uPA) and matrix metalloproteinase (MMP), which were demand for ECM degradation [36]. As expected, MMP-9 and uPA were elevated upon HGF stimulation. Simm530 significantly inhibited HGF-induced MMP expression and urokinase activity in a dose-dependent manner (Figure 4E and 4F).

Activation of c-Met drives a complex genetic program termed invasive growth, which is pivotal in driving cancer cell invasion and metastasis [37, 38]. *In vitro*, this morphogenetic program was replicated by stimulating cultured MDCK epithelial cells with HGF in 3D multicellular-branched morphogenesis model and 2D scattering assay [5, 39, 40]. We therefore chose these two representative models, cell scattering and morphogenesis, to evaluate the inhibitory effect of Simm530 on c-Met-driven invasive growth. As expected, MDCK cells performed cell-dissociation appearance and multicellular-branched structures upon HGF stimulation. However, Simm530 strongly inhibited cell scattering (Figure 4G) and morphogenesis (Figure 4H), indicating Simm530 inhibited HGF-induced c-Met-driven invasive growth. In accordance with the above observations, Simm530 effectively inhibited c-Met signaling in NCI-H441 and MDCK cells upon HGF stimulation (Figure 4I, 4J). Together, Simm530 inhibits HGF/c-Met axis and further inhibits HGF-stimulated cell metastatic behaviors.

### Simm530 significantly inhibits c-Met-driven tumor growth *in vivo*

**Figure 5:**
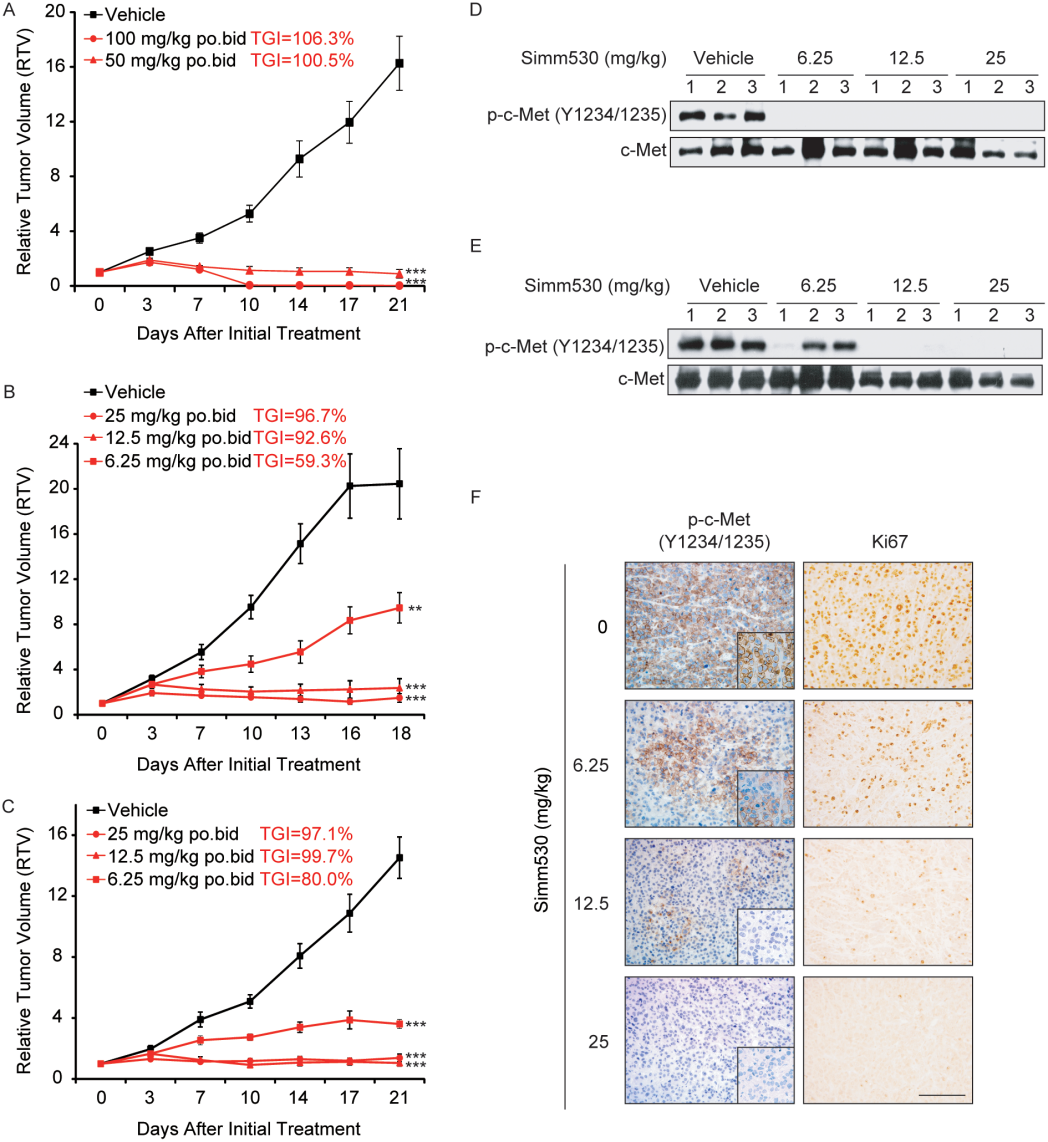
Simm530 inhibits c-Met-driven tumor growth *in vivo* **A**&**B.** Simm530 inhibited tumor growth in EBC-1 xenografts. Simm530 was administered orally twice daily after the tumor volume reached 100 to 200 mm^3^. The results are expressed as the mean ± SEM (n=6 per group). The percent tumor growth inhibition values (TGI) were measured on the final day of the study for the drug treated mice compared with the vehicle mice. Significant difference from the vehicle group was determined using one-way ANOVA, ** P < 0.01; *** P < 0.001. **C.** Simm530 inhibits tumor growth in SNU-5 xenografts. Simm530 was administered orally twice daily after tumor volume reached 100 to 200 mm^3^. The results are expressed as the mean ± SEM (n=6 per group). The percent tumor growth inhibition values (TGI) were measured on the final day of the study for the drug treated mice compared with the vehicle mice. Significant difference from the vehicle group was determined using one-way ANOVA, *** P < 0.001. **D**&**E.** Immunoblotting analysis of phospho-c-Met was determined in the EBC-1 (D) and SNU-5 (E) xenograft model, at 2 h after final administration of Simm530. **F.** An IHC evaluation of phospho-c-Met and Ki67 expression was determined in SNU-5 xenograft model, at 2 h after final administration of Simm530 (scale bars, 100 μm).

Encouraged by the potency of Simm530 in reversing c-Met-stimulated neoplastic phenotypes *in vitro*, we proceeded to evaluate its antitumor efficacy *in vivo*. Two representative tumor xenograft models driven by dysregulated c-Met were chosen: An EBC-1 human NSCLC xenograft model and a SNU-5 human gastric carcinoma xenograft model. In EBC-1 xenograft model, oral administration of Simm530 twice daily produced dose-dependent antitumor activity (Figure 5A, 5B). Tumor stasis was observed even at the dose of 12.5 mg/kg. Furthermore, Simm530 caused tumor regression at the dose of 50 and 100 mg/kg, and 4 of the 6 mice showed no signs of tumor at the 100 mg/kg twice daily dose (Figure 5A, 5B). Simm530 was well tolerance (lack of significant weight loss) at all tested dosages (data not shown). Similar results were obtained in SNU-5 xenograft model. Simm530 caused tumor stasis and regression at doses of 12.5 mg/kg and 25 mg/kg (Figure 5C).

We also evaluated the intratumoral Ki67 proliferation index and observed a significant decrease in the group treated with Simm530. In agreement with the suppressed tumor growth, a marked and dose-dependent inhibition in the intratumoral phosphorylation of c-Met was observed at 2 hours after the final dosage (Figure 5D, 5E, and 5F). Taken together, Simm530 showed a robust antitumor efficacy that was correlated with the inhibition of c-Met activation in c-Met-driven tumor models.

### Simm530 inhibits c-Met-mediated angiogenic effects *in vitro* and *in vivo*

**Figure 6:**
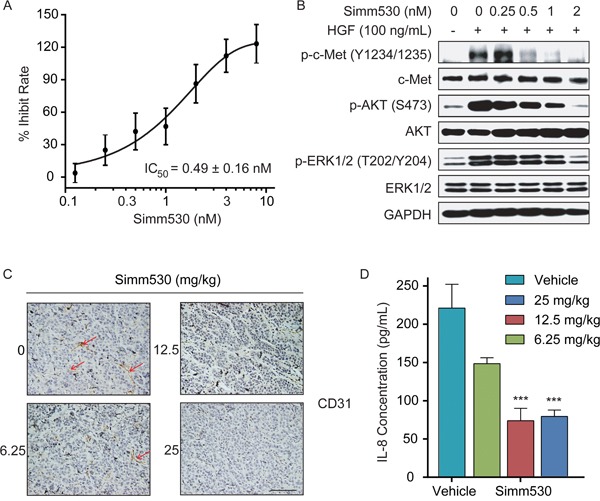
Simm530 inhibits c-Met-mediated angiogenic effects *in vitro* and *in vivo* **A**&**B.** Simm530 inhibited HGF-stimulated primary HUVEC cells proliferation (A) and c-Met signaling (B). Pre-starved primary HUVEC cells were treated with increasing concentration of Simm530 and HGF (100 ng/mL)for 24 h. Cell viability was measured by CCK-8 assay. Pre-starved primary HUVEC cells treated with Simm530 for 2 h, following 100 ng/mL HGF stimulation for 15 minutes were then lysed and subjected to immunoblotting analysis. Representative data are shown from three independent experiments. **C.** An IHC evaluation of CD31 expression was determined in EBC-1 xenograft model, at 2 h after final administration of Simm530 (scale bars, 100 μm). **D.** Simm530 reduced secretion of human IL-8 in SNU-5 xenograft model. Serum levels of human IL-8 were determined by ELISA assay of SNU-5 xenografts on day 21. The results are expressed as the mean ± SD. *** P < 0.001 vs vehicle group, determined using ANOVA test.

HGF/c-Met pathway promotes angiogenesis, which is also an important process in tumor formation and metastasis [9–11]. HGF/c-Met signaling is a potent direct inducer of endothelial cell growth and promotes angiogenesis. Hence, we assessed the anti-angiogenesis potential of Simm530. As shown in Figure 6A, Simm530 inhibited HGF-stimulated proliferation of HUVEC cells in a dose-dependent manner, with an IC_50_ value of 0.49 ± 0.16 nM. Consistently, c-Met phosphorylation and its downstream signaling were potently inhibited by Simm530 in HGF-stimulated HUVEC cells (Figure 6B). The *in vivo* anti-angiogenic effect of Simm530 was assessed for intratumoral modulation of microvessel density (MVD) by immunostaining for platelet endothelial cell adhesion molecule 1 (CD31) in the xenograft model. A significant reduction of CD31-positive endothelial cells was observed upon Simm530 treatment (Figure 6C). HGF/c-Met has also been shown to increase the secretion of pro-angiogenic factors by tumor cells, such as IL-8 [10, 41]. Therefore, the effect of Simm530 on IL- 8 plasma levels *in vivo* was also investigated. We found a significant reduction in IL-8 plasma level of Simm530 treated group (Figure 6D). All these results supported the conclusion that antitumor efficacy of Simm530 in part attributed to its anti-angiogenic activity.

## DISCUSSION

Aberrant c-Met activation promotes tumor formation and metastasis, as well as mediating resistance to approved therapies [12–15]. Therefore, inhibition c-Met signaling could have significant potential for the treatment of human cancers where the c-Met pathway is over-activated.

In fact, most c-Met inhibitors currently undergoing clinical trials are multi-target inhibitors, which may result in unwanted off-target toxicities [6, 16–22]. In this study, we reported a c-Met inhibitor, Simm530, featured with high selectivity against c-Met. Simm530 showed at least above 2,000-fold selectivity over a panel of 282 human kinases, making it one of the most selective c-Met inhibitors described to date. In accordance with this observation, cancer cells with low c-Met activity were markedly less sensitive (more than 50,000-fold) to Simm530 than c-Met-addicted cells. Since lacking the confounding issue of off-target kinase inhibition, Simm530 could specifically achieve the therapeutic potential of c-Met inhibition alone in human cancers addicted to c-Met aberrations. In addition, the feature of high selectivity makes Simm530 ideally suitable for use as a tool inhibitor in preclinical models to dissect the role of c-Met catalytic activity in cancer progression.

c-Met-driven invasive growth programme is an integrated set of cellular responses, including survival, proliferation, cell-cell dissociation, migration, invasion and ECM degradation. This complex programme functionally facilitates the primary transformed phenotype of tumor as well as the subsequent phases of neoplastic progression [6, 37, 38]. In this study, the formation of branching tubular structures and cell scattering of MDCK cells by HGF stimulation was significantly inhibited by Simm530. Consistently, reduction of c-Met-stimulated cell proliferation, migration, invasion and ECM degradation upon Simm530 treatment was obtained, and further confirming the potent inhibitory effect of Simm530 on c-Met-driven invasive growth. All these indicated that a potential role of Simm530 against tumor progression and metastasis.

HGF/c-Met system functions as a potent pro-angiogenic cue to exacerbate the malignant behavior of cancer cells. c-Met has been reported expressing in endothelial cells and HGF is able to directly stimulate endothelial cell growth and survival [6, 42]. c-Met and HGF could also promoted angiogenic factors secretion. Thus, c-Met inhibition is believed to give rise to potent anti-angiogenic effect apart from its direct devastating impact on tumor cells. Consistently, our data showed that Simm530 powerfully inhibited HGF-evoked HUVEC survival, intratumoral CD31 expression, as well as robustly decreased IL-8 secretion that correlated with its antitumor activity *in vivo*. Taken together, the present results suggested anti-angiogenic effect as an important mechanism-of-action in Simm530 antitumor efficacies.

In summary, Simm530 was identified as a novel, highly selective and potent c-Met inhibitor. It strongly inhibited c-Met phosphorylation and the downstream signaling across different oncogenic forms in c-Met over-activated cancer cells and endothelial cell. It exhibited potent antitumor activity via a mechanism of combined anti-proliferation and anti-angiogenic effects. The potent antitumor efficacy, high selectivity and potential broad therapeutic window of this molecule suggest its potential as a novel agent for treatment of c-Met-driven cancers.

## MATERIALS AND METHODS

### Compound

Simm530[6-(difluoro(6-(1-methyl-1H-pyrazol-4-yl)-[1,2,4]triazolo[4,3-b][1,2,4]triazin-3-yl)methyl)quinolone; Figure 1A] was synthesized at Prof. Wenhu Duan's Laboratory in Shanghai Institute of Materia Medica. The purity of Simm530 was 99%.

### Reagents

Recombinant human HGF was acquired from PeproTech Inc. The antibodies specific to phospho-c-Met (Y1234/1235), phospho-c-Met (Y1349), phospho-AKT (S473), phospho-ERK1/2 (T202/Y204), AKT, ERK1/2, Cyclin D1 and Cyclin E were purchased from Cell Signaling Technology; Antibodies against phosphotyrosine (PY99), c-Met, β-actin were purchased from Santa Cruz Biotechnology; Antibody against Ki67 was purchased from Epitomics Inc; Antibody against CD-31 was purchased from Abcam; Antibody against GAPDH was purchased from Kangcheng Bio.

### Cell culture

The following cells were purchased from American Type Culture Collection (Manassas, VA, USA), including NCI-H1993, NCI-H596, NCI-H441, NCI-H661, Calu-3, SNU-5, SNU-16, SNU-1, AGS, MDA-MB-231, HepG2, HT-29, HCT116, SK-MEL-28, and DU145. EBC-1, MKN-45 and NCI-H226 cells were purchased from Japanese Research Resources Bank (Tokyo, Japan). MDCK cells were a gift from Dr. H. Eric Xu of Shanghai Institute of Material Medical. BaF3 cell line was purchased from Deutsche Sammlung von Mikroorganismen und Zellkulturen GmbH (Braunschweig, Germany). SMMC-7721, ACHN, HMEC, NCI-H292, NCI-H358, NCI-H460, MDA-MB-435, T47D, MCF-7, BEL-7404, 786-O and U-87MG were obtained from Typical culture preservation commission cell bank, Chinese academy of sciences. Human gastric epithelial cell line GES-1 cells were obtained from Shanghai Cancer Institute, Renji Hospital, Shanghai Jiaotong University School of Medicine (Shanghai, China). SK-OV-3 was obtained from Fudan University (Shanghai, China). HUVEC was obtained from AllCells Bio (Shanghai, China). Cells were routinely maintained according to recommendations of their suppliers.

All the cell lines used in this study were obtained during March to August in 2011 and maintained in appropriate medium as suppliers suggested. All the cell lines were authenticated using STR (short tandem repeats) testing by Gene sky Biopharma Technology (Shanghai, China) (last tested in 2015).

### DNA plasmids construction, virus production and infection

The retroviral constructs pBABE-TPR-MET, empty vectors and c-Met variant 2 *(METV2)* cDNA were obtained from Addgene (Cambridge, MA). pBABE-METV2 was constructed using recombinant polymerase chain reaction and subsequently was sub-cloned into the pBABE-puro vector. pBABE-METV2 mutants were constructed with a site-directed mutagenesis kit (Sbsbio, Shanghai, China). To generate cells with stable expression, the plasmids were transfected into amphotropic phoenix 293T packaging cells with Lipofectamine 2000 (Invitrogen, Grand Island, NY). After 48 h, virus-containing medium was collected, filtered, and used to infect host cells in the presence of 6 mg/ml of polybrene. The stable transfectants were obtained by selection with 1 mg/mL puromycin (Sigma, St. Louis, MO) for two weeks followed by immunoblotting validation.

### c-Met small interfering RNA and transfection

MISSION^®^siRNA against c-Met and RNAi Negative Control were obtained for Sigma for siRNA experiments, 6×10^5^ MKN-45 cells were seeded in 6-well plates. After 24 h, cells were transfected with siRNAs with Oligo fectamine RNAimax reagent (Invitrogen) according to the manufacturer's instructions. 24 h later, cell was treated with the Simm530 or DMSO vehicle control for 72 hours, and then cells were harvested for cell proliferation analysis or immunoblotting analysis.

### ELISA kinase assay

The kinase domain of c-Met was expressed using the Bac-to-Bac™ baculovirus expression system (Invitrogen, Carlsbad, CA, USA) and purified on Ni-NTA columns (QIAGEN Inc., Valencia, CA, USA). The tyrosine kinase activities of c-Met were evaluated according to the reported protocol [43]. Briefly, 20 μg/mL poly (Glu, Tyr)_4:1_ (Sigma, St. Louis, MO, USA) was pre-coated in 96-well plates as a substrate. A 50-μL aliquot of 10 μmol/L ATP solution diluted in kinase reaction buffer (50 mmol/L HEPES [pH 7.4], 50 mmol/L MgCl_2_, 0.5 mmol/L MnCl_2_, 0.2 mmol/L Na_3_VO_4_, and 1 mmol/L DTT) was added to each well; 1 μL of various concentrations of indicated compound diluted in 1% DMSO (v/v) (Sigma) were then added to each reaction well. DMSO (1%, v/v) was used as the negative control. The kinase reaction was initiated by the addition of kinase diluted in 49 μL of kinase reaction buffer. After incubation for 60 min at 37°C, the plate was washed three times with phosphate-buffered saline (PBS) containing 0.1% Tween 20 (T-PBS). Anti-phosphotyrosine (PY99) antibody (100 μL; 1:500, diluted in 5 mg/mL BSA T-PBS) was then added. After a 30-min incubation at 37°C, the plate was washed three times, and 100 μL horseradish peroxidase-conjugated goat anti-mouse IgG (1:2000, diluted in 5 mg/mL BSA T-PBS) was added. The plate was then incubated at 37°C for 30 min and washed 3 times. A 100-μL aliquot of a solution containing 0.03% H_2_O_2_ and 2 mg/ml o-phenylenediamine in 0.1 mol/L citrate buffer (pH 5.5) was added. The reaction was terminated by the addition of 50 μL of 2 mol/L H_2_SO_4_ as the color changed, and the plate was analyzed using a multi-well spectrophotometer (SpectraMAX 190, Molecular Devices, Sunnyvale, CA, USA) at 490 nm. The inhibition rate (%) was calculated using the following equation: [1-(A490/A490 control)] × 100%. The IC_50_ values were calculated from the inhibition curves in two separate experiments. For ATP competitive assay, various concentrations of ATP were diluted for the kinase reaction. The results were analyzed in Lineweaver-Burk plots.

### Kinase profiling

The activity of Simm530, at a concentration of 1 μmol/L using an ATP concentration of 10 μM, was screened against a protein kinase panel of 282 human protein kinases by Eurofins using the Eurofins Kinase Profiler Selectivity Testing Service.

### Immunoblotting

Cells were cultured under regular growth conditions to exponential growth phase. Then the cells were treated with Simm530 for indicated time and lysed in 1×SDS sample buffer. If HGF treatment was required, cells were starved in serum-free medium for 24 h, and followed by treatment with Simm530 plus recombinant human HGF for appropriate time. Those cell lysates were subsequently resolved on 10% SDS-PAGE, and transferred to nitrocellulose membranes. Proteins were probed with specific antibody then subsequently with secondary horseradish peroxidase-conjugated antibody. Finally, immunoreactive proteins were detected using an enhanced chemiluminescence detection reagent (Pierce, Thermo Fisher Scientific Inc.).

### Cell proliferation assay

Cells were seeded in 96-well tissue culture plates. On the next day, cells were exposed to various concentrations of compounds and further cultured for 72 h. For Human umbilical vascular endothelial cells (HUVEC) (passage 3) were transferred to basal medium (without growth factor and serum) for 24 h before compounds treated for another 24 h. Finally, cell proliferation was determined using sulforhodamine B (SRB) assay, Thiazolyl Blue Tetrazolium Bromide (MTT; Sigma) assay or Cell Counting Kit (CCK-8) assay. IC_50_ values were calculated by concentration-response curve fitting using a SoftMax pro-based four-parameter method.

### Cell cycle analysis

The effects of Simm530 on cell cycle progression and population distribution were determined by flow cytometry. Cells were seeded at 2 × 10^5^ cells in 6-well plates and treated with Simm530 at indicated concentration or vehicle as a control. After 24 h, cells were collected, fixed and stained with propidium iodide (10 μg/mL) for 30 min, then analyzed using a flow cytometer (FACS Caliber instrument; Becton, Dickinson & Co.). Data were plotted using CellQuest (Becton, Dickinson &Co).

### Migration and matrigel invasion assays

For the migration assay, NCI-H441 cells suspended in serum-free medium (1.5×10^5^ cells per well) were seeded in 24-well Transwell plates (pore size, 8 μm; Corning). The bottom chambers were filled with serum-free medium supplemented with HGF (100 ng/mL), and 0.8, 4, 20 and 100 nM of Simm530 was added to both sides of the membrane. The cultures were maintained for 24 h, followed by the removal of non-motile cells at the top of the filter using a cotton swab. The migrating cells were fixed in paraformaldehyde (4%) and stained with crystal violet (0.1%) for 15 min at room temperature. The dye that was taken up by the cells bound to the membrane was released by the addition of 100 μL 10% acetic acid, and the absorbance of the resulting solution was measured at 595 nm using a multiwell spectrophotometer (SpetraMAX 190, from Molecular Devices, Palo Alto, CA, USA). The assay was performed in triplicate. Images were obtained using an Olympus BX51 microscope.

For the invasion assay, NCI-H441 cells were cultured in the top chambers containing Matrigel-coated membrane inserts (Matrigel, BD). The ensuing procedure was identical to the migration assay. The assay was performed in triplicate. Images were obtained using an Olympus BX51 microscope.

### Gelatin zymography

NCI-H441 cells were seeded in a six-well plate (5×10^5^ per well) and incubated at 37°C. After starved for 24 h, cells were incubated in serum-free medium supplemented with or without HGF (100 ng/mL) in the presence of various concentrations of Simm530 for another 24 h. The conditioned medium was collected and centrifuged at 12,000 g for 15 min and concentrated by Micro centrifugal filter device (Millipore Corporation). Then the supernatants were collected and separated on 7.5% SDS-PAGE with 2 mg/mL gelatin incorporated into the gel mixture. Following electrophoresis at 4°C, the gels were rinsed briefly with distilled water and washed three times (15 min each) with 150 ml of 2.5% Triton X-100 solution on a rotary shaker. The gels were then incubated at 37°C for 18 h in 250 ml of 50 mmol/L Tris·HCl (pH 7.5) that contained 10 mmol/L CaCl_2_, 1 μM ZnCl_2_, 1% Triton X-100, and 0.02% NaN_3_. After incubation, the gels were stained with 100 ml of 50% methanol, 10% acetic acid, and 0.1% Coomassie blue R-250 for 3 h, then destained with 50% methanol and 10% acetic acid. Finally, the gels were immersed in distilled water for 20 min and quantified by densitometric analysis using Image Lab software (Bio-Rad Laboratories, Inc.).

### Urokinase-type plasminogen activator (uPA) assay

MDCK cells (1.5-2 ×10^3^ cells per well) were plated in 96-well plate and grown overnight. Various concentrations of Simm530 and 100 ng/mL HGF were added to the appropriate wells and incubated for 24 h. The plate was processed for determination of plasminogen activity by first rinsing wells twice with DMEM (without phenol red), then adding 200 μL of reaction buffer [10%(v/v) 3 mM chromozym PL (Roche) in 100 mM glycine, 40%(v/v) 50 mM Tris (pH 8.2), 50%(v/v) 0.05 unit/mL plasminogen (Roche) in DMEM (without phenol red), and 0.2% Tween-20] to each well. After incubation at 37°C, 5% CO_2_ for 6 h, the absorbance of each well was read at 405 nm.

### Scattering assay

MDCK cells (1.5×10^3^ cells per well) were plated in 96-well plates and grown overnight. Increasing concentrations of Simm530 and HGF (100 ng/mL) were added to the appropriate wells, and the plates were incubated at 37°C and 5% CO_2_ for 24 h. The cells were fixed with 4% paraformaldehyde for 15 min at room temperature and then stained with 0.2% crystal violet. The assay was performed in triplicate. Images were obtained using an Olympus IX51 microscope.

### Cell branching morphogenesis

MDCK cells at a density of 2×10^4^ cells/mL in DMEM medium were mixed with an equal volume of collagen I solution and plated at 0.1 mL/well in a 96-well culture plate. After incubation for 1-2 h at 37°C and 5% CO_2_ to allow the collagen to gel, HGF (100 ng/mL) with or without Simm530 at various concentrations dissolved in 100 μL of growth medium was added to each well. The medium was replaced with fresh growth medium every 2 days. Images were obtained using an Olympus IX51 microscope after 5 days.

### *In vivo* antitumor activity studies

Female nude mice (4-6 weeks) were housed five or six mice per cage in a specific pathogen-free room with a 12 h light/dark schedule at 25°C ± 1°C and were fed an autoclaved chow diet and water ad libitum. Animal procedures were performed according to institutional ethical guidelines of animal care. The SNU-5 or EBC-1 cells at a density of 5 ×10^6^ in 200μL were injected s.c. into the right flank of nude mice and then allowed to grow to 700-800 mm^3^, defined as a well-developed tumor. After that, the well-developed tumors were cut into 1 mm^3^ fragments and transplanted s.c. into the right flank of nude mice using a trocar. When the tumor volume reached 100-200 mm^3^, the mice were randomly assigned into vehicle and treatment groups (n = 6 per group). Vehicle groups were given vehicle alone, and treatment groups received Simm530 at indicated doses via p.o. administration twice daily for indicated days. The sizes of the tumors were measured twice per week using microcaliper. The tumor volume (TV) was calculated as: TV = (length×width^2^)/2 and the individual relative tumor volume (RTV) was calculated as follows: RTV = V_t_/V_0_, where V_t_ is the volume on each day, and V_0_ is the volume at the beginning of the treatment. RTV was shown on indicated days as the median RTV ± SE indicated for groups of mice.

Percent (%) inhibition values (TGI) were measured on the final day of study for drug-treated compared with vehicle-treated mice and are calculated as 100× {1−[(V_Treated Final day_−V_Treated Day 0_) / (V_Control Final day_ − V_Control Day 0_)]}.

### Immunohistochemistry

Tumor specimens were fixed in 4% paraformaldehyde for 24 h. Tumor samples were subsequently paraffin-embedded, and sliced onto microscope slides. After dewaxing and blocking endogenous peroxidase activity with 3% H_2_O_2_, the sections were incubated with 1.5% normal goat serum, followed by overnight incubation at 4°C with anti-phospho-c-Met antibody (Y1234/1235), anti-CD31 antibody or anti-Ki67 antibody. Then, the sections were incubated with biotin-conjugated anti-rabbit IgG for 2 h at 37°C followed by incubating for 1 h with avidin-biotin-peroxidase complex (ABC) using a Vectastain ABC kit(Vector Laboratories). Staining was detected using the DAB (3, 3′-diaminobenzidine tetrahydrochloride) Liquid System (ZSGB-Bio) and imaged in 5 different fields of each section. Anti-phospho-c-Met and anti-CD31 antibody immunostained sections were counterstained using hematoxylin.

### Human IL-8 immunoassay

Serum levels of human IL-8 were detected by using Human IL-8 Quantikine ELISA Kit (R&D Systems, Inc.) follows the instructions.

### Statistical analysis

Between group differences were analyzed by one-way ANOVA, with P-values <0.05 for overall comparisons tested by post hoc pairwise comparisons using the Tukey multiple comparison tests. All statistical analyses were performed using R Ver. 3.0.2.

## SUPPLEMENTARY FIGURES AND TABLE


