# Supplementary material for: Simm530, a novel and highly selective c-Met inhibitor, blocks c-Met-stimulated signaling and neoplastic activities
Source: Oncotarget. 2016 May 13;7(25):38091–104. doi: 10.18632/oncotarget.9349 (PMC5122374; doi:10.18632/oncotarget.9349)
Supplement: Supplementary file 1 [file oncotarget-07-38091-s001.pdf]

SUPPLEMENTARY FIGURES AND TABLE

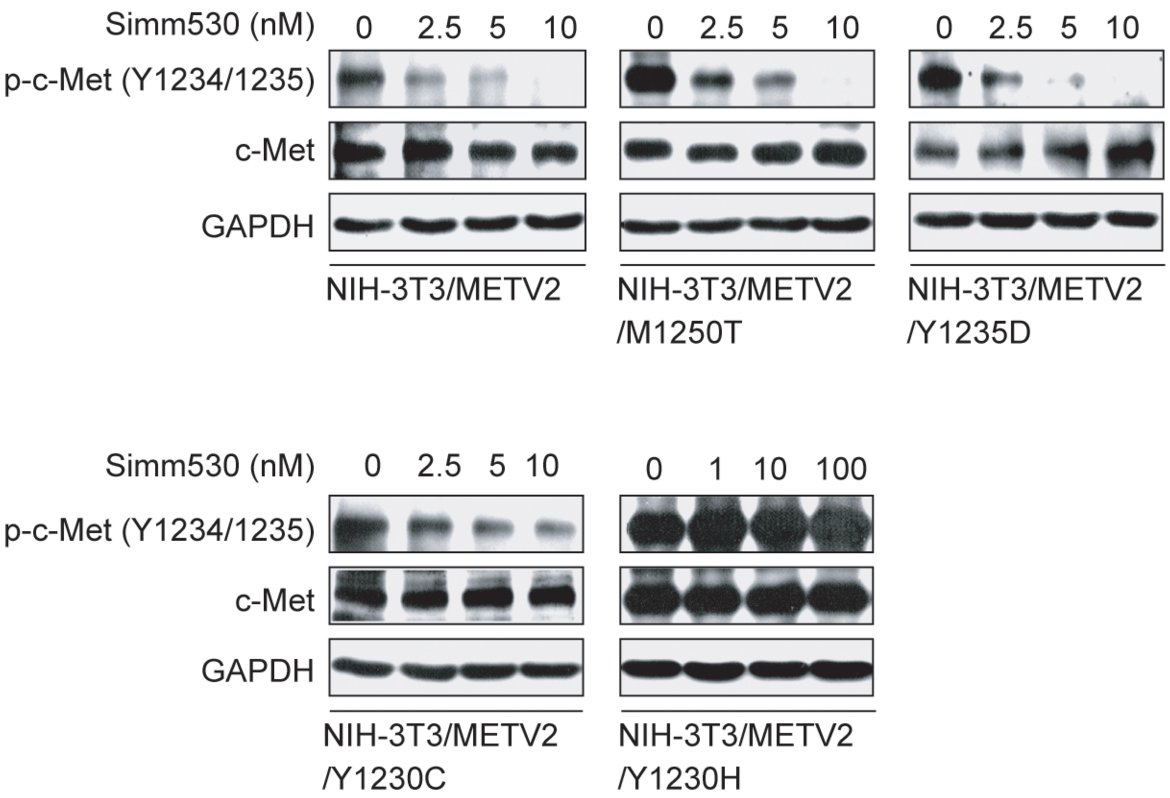

Supplementary Figure S1: Effect of Simm530 on c-Met phosphorylation in NIH-3T3 cell lines expressing the respective c-Met mutants. Cells were treated with increasing concentrations of Simm530 for 2 h. Then, cells were lysed and subjected to Western blot analysis.

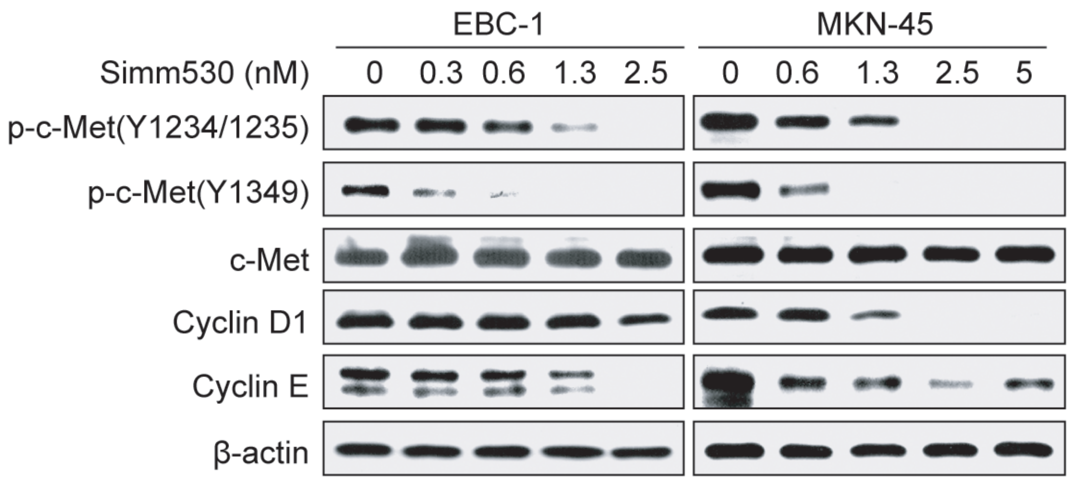

Supplementary Figure S2: EBC-1 and MKN-45 cells were treated with Simm530 for 24 h, and indicated G_0/G_1-S phase regulated proteins were analyzed by immunoblot.

Supplementary Table S1: Anti-proliferativeactivity of Simm530 on c-Met-addicted cell lines

Simm530	IC_{50} (nM)
EBC-1	0.7±0.2
NCI-H1993	0.8±0.3
SNU-5	0.9±0.2
MKN45	0.7±0.1
BaF3/TPR-Met	1.1±0.3

IC_{50} values were shown as mean ± SD.
